# Radiation Risk Analysis of Neuropsychiatric Disorders in Ukrainian Chornobyl Catastrophe Liquidators

**DOI:** 10.3389/fpsyt.2020.553420

**Published:** 2020-11-17

**Authors:** Konstantyn N. Loganovsky, Sergii V. Masiuk, Vladimir A. Buzunov, Donatella Marazziti, Yuliya S. Voychulene

**Affiliations:** ^1^State Institution “National Research Center for Radiation Medicine of the National Academy of Medical Sciences of Ukraine”, Kyiv, Ukraine; ^2^Dipartimento di Medicina Clinica e Sperimentale Section of Psychiatry, University of Pisa, Pisa, Italy

**Keywords:** chornobyl disaster, ionizing radiation, neuropsychiatric disorders, radiation risk analysis, liquidatord

## Abstract

**Goal:** To explore the possible impact of ionizing radiation in the pathophysiology of neuropsychiatric disorders amongst clean-up workers of the Chornobyl catastrophe (liquidators).

**Design, object, and methods:** Retrospective-prospective study (1987–2015) of liquidators from the State Register of Ukraine (SRU) with radiation doses records and Clinical-Epidemiological Register (CER) of the State Institution ≪National Research Center for Radiation Medicine of the National Academy of Medical Sciences of Ukraine≫ (NRCRM). Moreover, cohort and cross-sectional studies of the randomized sample of liquidators from the CER (exposed group, 198 subjects) were examined. Internal control group included the liquidators irradiated in doses <50.0 mSv (42 persons). All subjects were assessed by a detailed clinical examination and a battery of standardized neuropsychiatric scales, psychometric, and neuropsychological tests. Descriptive and variation statistics, non-parametric criteria, regression-correlation analysis, survival analysis by Kaplan & Meier, and risk analysis were used.

**Results:** Exposed group vs. control group showed cognitive disorders in 99 (50.0%) vs. 20 (18.1%), (*P* = 0.04); affective disorders in 96 (48.3%) vs. 36 (32.7%) (*P* = 0.007), and stress-related disorders in 115 (58.4%) vs. 8 (7.3%) (*P* < 0.001). In the main group exposed to ≥50 mSv vs. internal control group (exposed to <50 mSv), affective disorders were present, respectively, in 89 (56.4%) vs. 7 (19.1%) (*P* < 0.001), and stress-related disorders in 98 (62.8%) vs. 17 (40.4%) (*P* = 0.009). Relative risks (RR) and 95% confidential intervals (95%CI) of Incidence of some neuropsychiatric disorders in liquidators of 1986–1987 related to internal control (doses <50 mSv) were as follows: organic psychosis (RR = 3.15; 95% CI: 2.6; 3.7); non-psychotic organic brain damage (RR = 1.99; 95% CI: 1.6; 2.5); acute (RR = 1.40, 95% CI: 1.3; 1.5), and chronic cerebrovascular disorders (RR = 1.23; 95% CI 1.0;1.5). Neuropsychiatric diseases show a strong, increasing, and approximately quadratic statistically significant (*Pv* < 0.001) relationship with individual dose, yielding an estimated excess relative risk ERR = 2.76 Sv^−2^ (95% CI 1.06–7.15).

**Conclusions:** Liquidators have an excess of cognitive, affective, and stress-related disorders. The risk of diseases rises with radiation dose. Radiation risks are revealed for organic psychoses, non-psychotic organic brain damage, acute and chronic cerebrovascular pathology.

## Introduction

The Chornobyl disaster was a catastrophic nuclear accident that occurred on the 26th April 1986 at the No. 4 nuclear reactor in the Chornobyl Nuclear Power Plant (ChNPP), near the city of Pripyat in the north of Ukraine (110 km from Kyiv), and it was the most serious accident ever to occur in the nuclear power industry. According to the International Nuclear and Radiological Event Scale (INES) the Chornobyl catastrophe was the highest, the 7th, level “major accident,” resulting in widespread health and environmental effects requiring implementation of planned and extended countermeasures (1). To date, there have been two accidents of this kind: the Chornobyl catastrophe (2) and Fukushima Daiichi nuclear disaster, a series of events beginning on 11 March 2011 (3, 4). In any case, radiological impact on people and environment of the Chornobyl catastrophe is around 10 times higher than that following the Fukushima Daiichi accident.

According to the latest estimates (5), ~1.8 EBq ^131^I radioactivity was thrown out of the destroyed ChNPP Unit, significantly higher than that following the Fukushima disaster. The same is concerning external doses of exposure: in Chornobyl the averaged dose for clean-up workers (“liquidators,” 600,000 persons) is assessed to be around 100 mSv (2, 6); for evacuees (135,000 persons) 33 mSv, for strict control zone inhabitants (living in the territories with radioactivity deposition >555 kBq·m^−2^) 50 mSv, and for low exposed (5,000,000 persons) 10–20 mSv (2, 6). On the contrary, the highest doses for Fukushima rescue workers were assessed to be 10–50 mSv, for Fukushima population 1–10 mSv, for Japan 0.1–1 mSv per year (3). For comparison, worldwide annual exposure to natural radiation sources would generally be expected to be in the range 1–10 mSv, with 2.4 mSv being the present estimate of the central value (7).

There are very contradictive assessments of the Chornobyl catastrophe health effects. The International, UN-associated, organizations (International Atomic Energy Agency (IAEA), World Health Organization (WHO), and the United Nations Scientific Committee on the Effects of Atomic Radiation (UNSCEAR) recognized only 31 death cases directly related to the accident, 134 verified cases of Acute Radiation Sickness (ARS) and around 6,000 cases of thyroid cancer reported in children and adolescents who were exposed at the time of the accident. They came to the conclusion that there was no sufficient scientific evidence concerning any other cancer and non-cancer Chornobyl health effects, while cataracts, leukemia, and cardiovascular (including cerebrovascular and neurocognitive) diseases are considered to be at the frame of radiation-associated effects in liquidators. Although those most highly exposed individuals are at an increased risk for radiation-associated effects, the great majority of the population is not likely to experience severe health consequences as a result of radiation from the Chornobyl accident. Many other health problems have been noted in the victims/survivors that were not attributed to radiation exposure, but to psychological consequences only. Therefore, in general, the conclusions of those agencies are as follows: the ChNPP accident was a tragic event for its victims, and those most affected suffered major hardship. Some people who dealt with the emergency lost their lives. Although those exposed as children, and the emergency and recovery workers, are at increased risk of radiation-induced effects, the vast majority of the population should not be concerned by serious health consequences. For the most part, individuals were exposed to radiation levels comparable to or just a few times higher than annual levels of natural background, and future exposures continue to slowly diminish as the radionuclides decay. Although lives had been seriously disrupted by the Chornobyl accident, from a radiological point of view, positive prospects for the future health of most individuals should prevail (2, 6, 8–10).

The UN Chornobyl forum (2006) concluded about persistent mental health worsening of the Chornobyl accident survivors as a result of (1) stress-related disorders; (2) effects on the developing brain; (3) organic mental disorders in liquidators, and (4) suicides. The revealed excess of cardiovascular and cerebrovascular disorders in liquidators was recognized as needing further investigation (8, 9).

By contrast, at the same time, ecologically oriented publications (Green Peace-related, (11–14)) argued that a so-called “atomic lobby” underestimates the environmental and health effects of the Chornobyl catastrophe. Green Peace is positive about the dramatic excess of cancer and non-cancer morbidity and mortality of the Chornobyl victims due to radiation exposure (11–14). However, it is likely that the truth seems to be in the middle (15–17).

There are few well-designed systematic epidemiological studies of mental health following the Chornobyl catastrophe. There is a consensus about long-term mental health deterioration, mainly at subclinical levels, attributed to stress and other non-radiation factors in residents of radioactively contaminated territories (18, 19), and cleanup workers, such as depression, anxiety and posttraumatic stress disorder (PTSD), suicidal ideation or attempted, or completed suicides (20, 21), alcohol abuse, poorer perceptions regarding personal physical and mental health (22). However, all these studies had no dosimetric support, with the exception of self-report exposure estimation (22). Therefore, the assessment of the radiation risk analysis for neuropsychiatric disorders in Chornobyl liquidators was impossible, which is why the attribution of all of these disorders to stress alone has no scientific base.

Interestingly, similar data on long-term mental health deterioration were reported after the A-bombing in Hiroshima and Nagasaki, Japan, 1945 (23, 24), Three Mile Island crisis, USA, 1979 (25, 26), and the Fukushima Daiichi accident, Japan, 2011 (27, 28).

Although a considerable worsening of mental health in the Chornobyl catastrophe survivors has been documented, however, the role of ionizing radiation in mental health deterioration is still unclear. Therefore, the purpose of our study was to assess the possible role of ionizing radiation in the pathophysiology of neuropsychiatric disorders amongst cleanup workers of the Chornobyl catastrophe (liquidators).

## Design, Object, and Methods

### Study Participants

We combined two sets of information:

1. *Follow-up data* collected between 1987 and 2015 from the State Register of Ukraine (SRU) with doses of exposure records involving 68,145 liquidators, and from the Clinical-Epidemiological Register (CER) of the State Institution ≪National Research Center for Radiation Medicine of the National Academy of Medical Sciences of Ukraine≫ (NRCRM) including 3,548 persons. Two diagnostic criteria for nervous and mental/behavioral disorders were used, according to the 9th and 10th editions of the WHO International Classification of Diseases (ICD-9 and ICD-10). Clinical neurological and psychiatric diagnosis for the registers SRU and CER were provided by certified neurologists and psychiatrists according to the current ICD diagnostic criteria at the time of examination. Further, harmonization between ICD-9 and ICD-10 diagnosis of neuropsychiatric disorders were done according to elaborated in NRCRM converting tables from ICD-9 toward ICD-10 neuropsychiatric disorders. The epidemiological methods, including risk-analysis, were used.
2. *Retrospective-prospective (1987–2018) cohort and cross-sectional cohort data following neuropsychiatric verification* with the external and internal control groups. In the Department of Radiation Psychoneurology of the Institute for Clinical Radiology (ICR) of NRCRM, the randomized sample of the Chornobyl clean-up workers from the CER of the NRCRM (main group 198 persons) was examined, together with 43 individuals evacuated from the Chornobyl exclusion zone. These subjects have been under prospective medical surveillance across their lifespan during the post-accidental years (1987–2018). Their neuropsychiatric diagnoses have been verified as described below.

The *inclusion criteria* in the main group were as follows: (1) availability in the CER of NRCRM; (2) participation in the works of clean-up of the consequences of the Chornobyl disaster in 1986–1987; (3) availability of the records of radiation doses; (4) men.

The patients' age of the main group at the moment of the last survey was between 39 and 87 years (mean ± SD: 60.0–8.5), and that at the time of the Chornobyl catastrophe was between 18 and 56 (mean ± SD: 32.1.1 ± 7.6). The doses of the external exposure of the examined clean-up workers were within the range of 0.6–5900.0 mSv with an average arithmetic dose (mean ± SD) of 456.0 ± 760.0 mSv. The dose of exposure distribution of the examinees is shown in Table 1. The clean-up worker subgroup irradiated at doses of 0.6–50.0 mSv (*n* = 42) was designated as the internal control group in relation to the main clean-up worker group. Such a decision to consider those exposed <50.0 mSv were based on: (1) current knowledge that there are no clear evidence-based data on deterministic (tissue reactions) radiation effects at such an exposure, and (2) the similar social-psychological status/exposure to both irradiated <50.0 and ≥50.0 mSv persons for the control non-radiation confounding factors.

**Table 1:** Distribution of cleanup workers of the Chornobyl catastrophe by radiation doses of external exposure.

**Radiation doses range, mSv**	***N***	**Radiation dose (Mean ± SD), mSv**
<50 (0.6–50)	42	21.3 ± 16.4
50–100	27	68.6 ± 13.7
100–250	42	180.8 ± 42.3
250–500	45	334.9 ± 79.9
500–1,000	19	691.4 ± 144.0
>1,000 (1,000–5,900)	23	2183.9 ± 1084.1

The comparison group (*n* = 110) was randomly formed from out- and inpatients of the Radiation Psychoneurology Department of ICR of NRCRM. Their age was between 45 and 70 years (mean ± SD: 53.6 ± 5.3) at the moment of the survey, and between 18 and 39 (mean ± SD:24.7–4.7) that at the moment of the Chornobyl disaster. The inclusion criteria in the comparison group were as follows: (1) no involvement in any radiation emergencies, nuclear tests, and therapeutic exposure; (2) absence of the extra irradiation in relation toward the background radioactivity with the exception of the medical diagnostic radiological procedures and air flights; (3) men; (4) age comparable to the main group. The exclusion criteria were as follows: (1) non-compliance with any of the inclusion criteria for the comparison group and (2) involvement in multicentre clinical trials.

### Assessments for Cross-Sectional Study

The unified neuropsychiatric examination was carried out in accordance with the ICD-10 criteria (29), by using the methodical recommendations and clinical guidelines for diagnostics and verifying organic brain damage following radiation exposure as a result of the Chornobyl accident developed in the Department.

The following scales were used for diagnosis:

- the Expanded Disability Status Scale, EDSS (30);
- the Kurtzke Functional Systems Scores, KFS (31);
- the Brief Psychiatric Rating Scale (BPRS) (32, 33).

The following scales were employed for the qualitative and quantitative psychopathological assessment:

- the General Health Questionnaire (GHQ-28) to assessing somatoform symptoms, anxiety/insomnia, social dysfunction, and severe depression (34);
- the Zung Self-Rating Depression Scale (SDS) to measure depression (35);
- the Posttraumatic Stress Disorder (PTSD) questionnaires, the Impact of Events Scale (IES) (36) and Irritability, Depression, Anxiety (IDA) used to assess the agitation associated with PTSD (37);
- the Mini-Mental State Examination (MMSE) (38) for screening diagnosis of cognitive impairment.
- the Rey Auditory Verbal Learning Test (RAVLT) for evaluating memory functions (39).
- the Wechsler Adult Intelligence Scale (WAIS), adapted in 2012 by the IMATON (St. Petersburg) (40), to measure Intelligent Quotients (IQ). Premorbid (pre-emergency) intelligence quotients (pre-IQ) were calculated using the demographical-based regression equation by Gao et al. (41). The level of cognition was determined by the operational criteria of cognition based on the estimation of both the current IQ and the cognitive deficit after the Chornobyl catastrophe by the differences between premorbid (pre-emergency) and current IQ (42, 43).

Following the comprehensive clinical neuropsychiatric examination according to the Chapters G&F of the ICD-10 criteria, as well as the results of psychometric scales and tests just described, the expert neuropsychiatric conclusions were done and were further statistically estimated.

The study was conducted after obtaining written informed consent from each participant according to the Declaration of Helsinki (44).

### Statistical Analyses

For statistical data analysis the descriptive statistics, regression-correlation analysis by Pearson & Spearman, Kaplan-Meier survival analysis, multiple linear and quadratic regressions, relative risk analysis and odds ratios, non-parametric criteria, as well as the tools for the graphical analysis, and the results presentation were used. The verification of the statistical hypothesis regarding the data correspondence to the normal distribution was carried out using the Kolmogorov-Smirnov criteria, adjusted by Lilliefors and Shapiro-Wilkes. Excel 8.0 spreadsheets were used to collect, store, and analyze the data. Statistical analysis was performed by Statistica 10.0 (StatSoft) (45), SPSS Statistics 17.0 (46) and EPICURE (HiroSoft http://www.hirosoft.com/) (47).

*Risk analysis of following neuropsychiatric verification* was performed using the well-known epidemiological statistical software EPICURE. Before this risk analysis, the data were cross-classified by the radiation dose (<0.05; 0.05–0.1; 0.1–0.2; 0.2–0.4; 0.4–0.8; 0.8–1.6; >1.6 Sv), age at exposure, that is, age at April 26, 1986 (<25; 25–40; >40 years), attained age, that is, the age at the moment of the neuropsychiatric pathology finding or at the censorship time (<30; 30–40; 40–50; 50–60; >60 years), as well as by the presence (was or was not) of post-traumatic stress disorder (PTSD). Therefore, 137 liquidators were included in the risk analysis, where 115 (83.9%) of them had verified neuropsychiatric disorders. Herewith, one of the modules of this epidemiological package PEANUTS, designed for data processing in the Cox Proportional Hazards Model for Censored Data, was used. The parameters of the linear and quadratic Cox proportional models of relative risk (RR) were estimated by EPICURE (47–49).

(1) $$\begin{array}{l} \lambda_{L}=\lambda_{0,L}\left(t\right)\cdot \exp\left(\alpha_{L,i}\cdot s_{i}+\beta_{L,i}\cdot a_{i}+\gamma_{L,i}\cdot e_{i}\right)\left[1+\rho_{L}\cdot D\cdot \right], \end{array}$$

(2) $$\begin{array}{l} \lambda_{Q}=\lambda_{0,Q}\left(t\right)\cdot \exp\left(\alpha_{Q,i}\cdot s_{i}+\beta_{Q,i}\cdot a_{i}+\gamma_{Q,i}\cdot e_{i}\right)\left[1+\rho_{Q}\cdot D^{2}\right], \end{array}$$

where λ_*L*_, λ_*Q*_ are the neuropsychiatric morbidity rates in linear and quadratic risk models, respectively; λ_0,*L*_(*t*), λ_0,*Q*_(*t*), a basic risk at a time *t* that is not assessed; *s*_*i*_, *a*_*i*_, and *e*_*i*_ categorical variables determining the presence or absence of PTSD, as well as the age group and the age at exposure group in which a Chornobyl clean-up worker was; *D* is the exposure dose (Sv); α_*L,i*_, β_*L,i*_, γ_*L, i*_, α_*Q,i*_, β_*Q,i*_, γ_*Q,i*_, ρ_*L*_, ρ_*Q*_ are parameters evaluated as a result of risk analysis. The sense of the parameters ρ_*L*_ and ρ_*Q*_ is the excess relative risk (ERR) in linear (A) and quadratic (B) models, respectively.

## Results

According to the register's data (SRU), the *incidence* of *organic, including symptomatic, mental disorders (organic psychosis)* (ICD-9: 293.0–294.9: ICD-10: F00-F05; F06.0, F06.2) was 101.99 per 10,000 persons, and it showed the highest statistically significant (*Pv* < 0.001) relative risk (RR = 3.15; 95% CI: 2.6; 3.7) (Table 2).

**Table 2:** Relative risks (RR) of Incidence of neuropsychiatric disorders in clean-up workers of 1986–1987 related to internal control (doses <50 mSv) according to the register's data.

**Disease**	**RR (95% CI)**	***Pv***
Organic, including symptomatic, mental disorders (organic psychosis) *(ICD-9: 293.0*–*294.9: ICD-10: F00-F05; F06.0, F06.2)*	3.15 (2.6; 3.7)	*Pv* <0.001
Other mental disorders due to brain damage and dysfunction and to physical disease *(ICD-10: F06.32, F06.4-F06.7)* and Personality and behavioral disorders due to brain diseases, damage and dysfunction (non-psychotic organic brain damage) *(ICD-9: 310.0*–*310.9; ICD-10: F07.0, F07.8, F07.9)*	1.99 (1.6; 2.5)	*Pv* < 0.001
Acute cerebrovascular disorders (stroke) *(ICD-9: 430.0*–*436.9; ICD-10: I60.0*–*I66.0)*	1.40 (1.3; 1.5)	*Pv* < 0.001
Chronic cerebrovascular disorders and sequelae of cerebrovascular disease *(ICD-9: 438.0*–*439.9; ICD-10: I67, I69)*	1.23 (1.0; 1.5)	*Pv* = 0.05

Non-psychotic organic disorders *(ICD-9: 310.0*–*310.9; ICD-10: ICD-10: F06.32, F06.4-F06.7F07.0, F07.8, F07.9)* showed a RR=1.99 (Pv <0.001; 95% CI: 1.6; 2.5). These disorders included organic depressive, emotionally labile [asthenic], mild cognitive, anxiety, dissociative, as well as organic personality disorders.

Acute cerebrovascular disorders (strokes) had a RR = 1.40 (*Pv* < 0.001; 95% CI: 1.3; 1.5), and consequences/chronic cerebrovascular pathology, including cerebral atherosclerosis, hypertensive encephalopathy, and chronic cerebral ischemia, a RR = 1.23 (*Pv* = 0.05; 95% CI: 1.0; 1.5).

According to these previous findings, an expert assessment of neuropsychiatric disorders was carried out (Table 3). In comparison with the unexposed control group, liquidators showed significantly more cognitive disorders, in particular, mild cognitive impairment and dementia, and an increased percentage of affective disorders, mainly severe depression. The frequency of affective and stress disorders was significantly increased in clean-up workers exposed to doses ≥50 mSv, as compared to those exposed to lower doses, with a tendency toward a greater frequency of cognitive impairment (Table 4).

**Table 3:** Assessment of cognitive, affective, and stress-related disorders in the main group (clean-up workers) and the comparison group.

**Disorders**	**Main group, *n* = 198**	**Comparison group, *n* = 110**	***Pv***
Cognitive disorders (all)	99 (50.0%)	20 (18.1%)	*Pv* = 0.04
Mild cognitive impairment	79 (39.9%)	16 (14.5%)	*P* < 0.001
Dementia	20 (10.1%)	4 (3.6%)	*Pv*=0.03
Affective disorders (all)	96 (48.3%)	36 (32.7%)	*Pv* = 0.007
Mild	43 (21.6%)	32 (29.1%)	*Pv* = 0.15
Severe	53 (26.7%)	4 (3.6%)	*Pv* < 0.001
Stress-related disorders (all)	115 (58.4%)	8 (7.3%)	*Pv* < 0.001
Mild	–	8 (7.3%)	*Pv* < 0.001
Severe	17 (8.9%)	–	*Pv* = 0.002

**Table 4:** Assessment of cognitive, affective, and stress-related disorders in clean-up workers exposed to radiation in doses <50 mSv (internal control) vs. those exposed to ≥50 mSv.

**Disorder**	**<50 mSv *n* = 42**	**≥50 mSv, *n* = 156**	***Pv***
Cognitive disorders (all)	19 (45.8%)	80 (52.3%)	*Pv* = 0.47
Mild cognitive impairment	15 (36.3%)	64 (41.0%)	*Pv* = 0.53
Dementia	4 (9.5%)	16 (10.3%)	*Pv* = 0.58
Affective disorders (all)	7 (19.1%)	89 (56.4%)	*Pv* < 0.001
Mild	2 (4.8%)	41 (26.3%)	*Pv* = 0.001
Severe	5 (11.9%)	48 (30.8%)	*Pv*=0.009
Stress-related disorders (all)	17 (40.4%)	98 (62.8%)	*Pv* = 0.009
Mild	14 (33.3%)	84 (53.9%)	*Pv* = 0.02
Severe	3 (7.1%)	14 (8.9%)	*Pv* = 0.49

**Figure 1:**
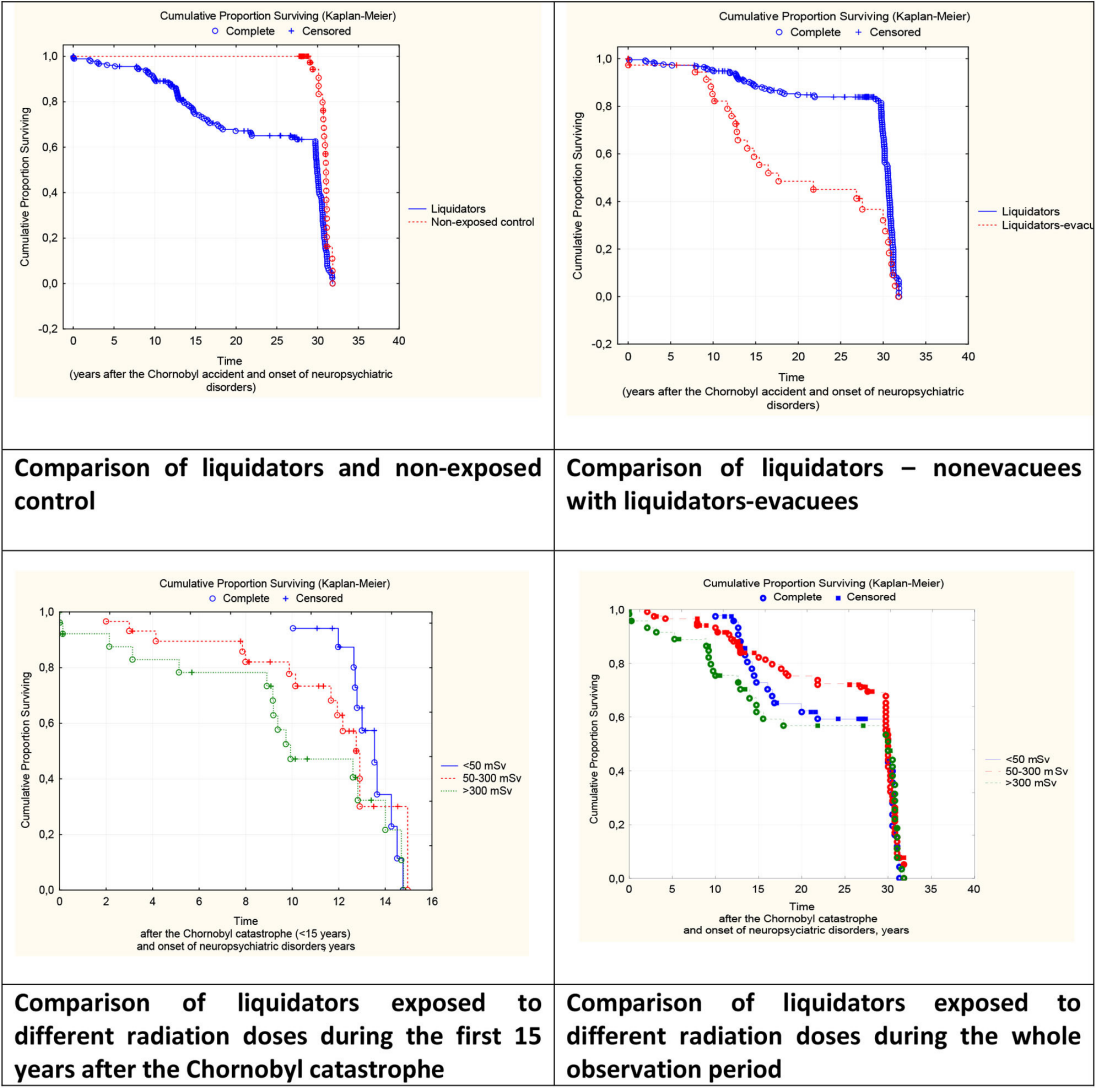
Survival curves (Kaplan & Meier) for neuropsychiatric pathology onset after the Chornobyl catastrophe.

According to the survival analysis, we considered the time of the onset of any neuropsychiatric pathology after the Chornobyl accident measured in years as the event in which the pathology arose after the catastrophe. As shown in Figure 1, neuropsychiatric disorders in clean-up workers appeared much earlier, that is to say, between 3 and 5 years after the disaster (Log-Rank Test = 4.96, *P* = 0.000). By contrast, only 30 years later, these disorders occurred approximately at same rate both in the clean-up workers and the unexposed control group. In addition, in the clean-up workers who were also evacuated from the Chornobyl exclusion zone, neuropsychiatric disorders began to emerge significantly later than 7–10 years after the disaster than in those not evacuated (Log-Rank Test = −3.13, *P* = 0.002). During the first 15 post-accidental years, the dependence of the occurrence of neuropsychiatric disorders upon the irradiation dose was detected at doses >300 mSv. The onset of these conditions occurred quite early, almost immediately after the disaster; at doses of 50–300 mSv 2 years later, and at doses lower than 50 mSv after ten 10 years (χ^2^ = 8.74 *P* = 0.01), this dependence disappears.

### Risk Analysis

Dose-response analysis for 137 liquidators was done with the help of PEANUTS module of EPICURE package. This module is used for analysis of ungroup censored survival data using partial likelihood methods for distribution-free hazard function, often called proportion hazards models or Cox proportional models (47–49). Odds ratio (OR) as well as parameters of the linear (A) and quadratic (B) model of relative risk were estimated.

**Figure 2:**
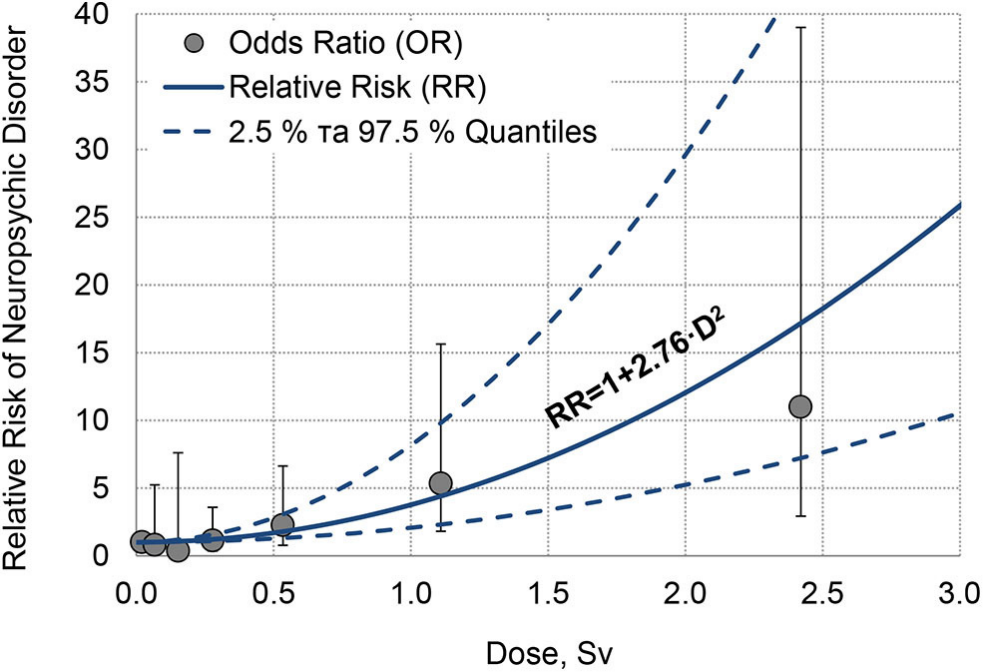
Dose-response dependence on neuropsychiatric disorders in liquidators.

The odds ratio for mental disorders and their 95% confidence interval, as well as the likelihood ratio test (LRT), were calculated for each group (Table 5). The overall risk of neuropsychiatric diseases statistically significant (*Pv* < 0.001) increases with the exposure dose. As seen in Table 5, the OR of neuropsychiatric diseases for persons with doses from 0.05 to 0.2 Sv is ever smaller than for the persons with dose <0.05 Sv (50 mSv) and for persons with doses from 0.2 to 0.4 Sv it is a bit higher, but the difference is not statistically significant. At the same time, for doses >0.4 Sv, the risk of neuropsychiatric diseases is 2–11 times larger compared with that at small doses (below 0.05 Sv), and such a difference is statistically significant. Also with high statistical significance (*Pv* < 0.001), the OR is on average 2.2 times larger for persons with post-traumatic stress disorder than for persons without it, and it is 3.5–11 times significantly larger (*Pv* < 0.001) for the persons who were older than 25 at the time of exposure than for the persons younger than 25. The overall risk of neuropsychiatric diseases statistically significantly decreases with the attained age (*Pv* < 0.001). This phenomena can be caused by a linear relation between attained age and time since exposure and just reflect the fact that the bulk of neuropsychiatric disorders in the Chornobyl clean-up workers realized during the first 15 years after the disaster (Figure 2).

**Table 5:** Odds ratio (OR), 95% confidence interval (CI), and reliability probability based on probability function (LRT) for the different groups of the clean-up workers.

**Parameter**	**Category**	**Number of cases**	**Total number**	**OR**	**95% CI**	**LRT**
Dose^a^, Sv	<0.05	22	27	1.00	—	*Pv* < 0.001
	0.05–0.1	11	15	0.80	0.12–5.23	
	0.1–0.2	11	16	0.36	0.01–7.60	
	0.2–0.4	31	35	1.13	0.35–3.57	
	0.4–0.8	17	20	2.25	0.76–6.63	
	0.8–1.6	8	9	5.32	1.80–15.6	
	1.6+	15	15	11.0	2.91–41.4	
PTSD^b^	No	44	57	1.00	—	*Pv* < 0.001
	Yes	71	80	2.18	1.40–3.46	
Age at exposure^c^, years	<25	26	27	1.00	—	*Pv* < 0.001
	25–40	71	91	3.46	1.91–6.26	
	40+	18	19	11.1	4.38–28.0	
Attained age^d^, years	<30	12	12	1.00	—	*Pv* < 0.001
	30–40	9	9	0.17	0.05–0.50	
	40–50	33	40	0.03	0.01–0.08	
	50–60	37	44	0.004	0.001–0.01	
	60+	24	32	0.001	0.0001–0.002	

a *Adjusted for attained age, PTSD, and age at exposure*;

b *adjusted for attained age, age at exposure, and dose*;

c *adjusted for attained age, PTSD, and dose*;

d *adjusted for age at exposure, PTSD, and dose*.

The estimation of the parameters in the dose-response linear model (A) yielded statistically significant (*Pv* < 0.001) excess relative risk ERR = 2.5 Sv^−1^ that falls within the 95% CI of 0.86–7.27. However, the Akaike information criterion (goodness of fit test based on the likelihood function) showed that the quadratic model (B) better fit the data. The estimation of the excess relative risk ERR in the quadratic model was 2.76 Sv^−2^ (*Pv* < 0.001) and falls within the 95% CI of 1.06–7.15. Figure 2 shows the quadratic dependence of the RR of the neuropsychiatric disease occurrence on exposure dose of the Chornobyl clean-up workers as well as the OR and 95% CI.

## Limitations

The present study suffers from some limitations that should be acknowledged. First, there exist different approaches in diagnosing mental disorders between the Western and Eastern countries, that is why we used ICD 9 and 10 criteria. Second, the dramatically social changes in post-soviet societies might have led to uncertainties in individual radiation doses estimation. Third, we did not control for possible confounding non-radiation factors. However, in spite of these limitations, we are of the opinion that our data are important in highlighting an evident brain damage following radiation exposure.

## Discussion

The main findings of our study represent a clear evidence of radiation-induced detrimental cerebral effect, as shown by the organic brain damage, or acute and chronic cerebrovascular pathology, depressive, and stress-related disorders amongst liquidators. The overall risk of neuropsychiatric pathology increases significantly with the irradiation dose and decreases with the attained age.

Our data are consistent with the excess and significant radiation risks for morbidity and mortality due to non-cancer radiation effects, mainly cardiovascular (including cerebrovascular) disorders in A-bomb- survivors (50–52), military radiochemical complex “Mayak” with the dramatic radiation accidents in the 50th of the XX Century (USSR, Russian Federation) (53, 54), other nuclear workers cohorts (55–58), and Chornobyl liquidators (59–62).

Therefore, the role of exposure to ionizing radiation in provoking brain effects, as a result of the Chornobyl accident is significant, especially at doses more 0.2–0.25 Sv of external irradiation, cannot be denied any longer.

Again, previous data also detected a radiation dose-dependent deterioration of the actual (current) cognitive functioning in comparison with premorbid (pre-radiation exposure) IQ due to the verbal IQ decrement was also revealed following the ARS as a result of the Chornobyl catastrophe (42), still in the liquidators as well, exposed to lower radiation doses (43), suggestive of a radiation-associated mild cognitive impairment (MCI). In addition, some characteristic post-radiation electroencephalographic (EEG) pattern (63) and neurophysiological radiation biomarkers according to quantitative EEG (qEEG) were found at exposure to radiation doses >1 Gy/Sv following ARS, so that the completely innovative neurophysiological (qEEG-based) biodosimeter has been proposed (64). Neurocognitive, neuropsychological, neurophysiological, neuroimaging, and neurovascular dose-related disturbances were revealed after exposure to ionizing radiation at doses >0.3 Gy/Sv (65–70). The changes in the amplitude-time parameters of the cognitive auditory evoked potentials, which dominate in the left fronto-temporal area, namely the Wernicke's cortical zone (71, 72), were detected in the Chernobyl clean-up workers. Based on the event-related potentials (ERP) research, the extreme radiosensitivity of the human brain was confirmed following irradiation even at the low doses, especially at the level of the cortical-limbic system in the dominant hemisphere and the Wernicke's area as well. The new dose-dependent effects for some brain changes in humans were estimated to occur at doses >0.05 Sv (71–74).

Although the pathophysiology of the brain effects of ionizing radiation is still unclear, some possible explanations have been proposed. They include, amongst others, inhibition of neurogenesis, mainly in the hippocampus, telomere length and gene expression changes, apoptosis, neuroinflammation, autoimmune processes, and glial mechanisms (75–81).

## Conclusions

The present study indicates that liquidators suffered from an excess of cognitive, affective and stress-related disorders. The risk of disease rises with dose of exposure. Their radiation risks for organic psychoses, non-psychotic organic brain damage, acute, and chronic cerebrovascular pathology were similarly high. It is disappointing that clinical and epidemiological studies with international expertise on the assessment of the neuropsychiatric effect of the Chornobyl disaster together with dosimetric support still need to be done. We are of the opinion that liquidators should be followed along their lifespan together with their offspring, in order to deepen our knowledge on radiation health effects and to improve radiation protection and safety for the next generations.

## Data Availability Statement

The datasets presented in this study can be found in online repositories. The names of the repository/repositories and accession number(s) can be found in the article/supplementary material.

## Ethics Statement

The studies involving human participants were reviewed and approved by Institutional Research Body, State Institution “National Research Center for Radiation Medicine of the National Academy of Medical Sciences of Ukraine”, Kyiv, Ukraine. The patients/participants provided their written informed consent to participate in this study.

## Author Contributions

All authors: design, planning, analyses, writing, references search, editing, and revision.

## Conflict of Interest

The authors declare that the research was conducted in the absence of any commercial or financial relationships that could be construed as a potential conflict of interest.
